# EGPA at Diagnosis: A Comprehensive Single-Center Profile of Laboratory Findings and Clinical Evidence

**DOI:** 10.3390/ijms27167116

**Published:** 2026-08-08

**Authors:** Silvia Brunetto, Francesca Dimasi, Cristiano Maiolo, Emanuela Zumbo, Federica Buta, Sebastiano Gangemi, Luisa Ricciardi

**Affiliations:** Department of Clinical and Experimental Medicine, Allergy and Clinical Immunology Unit, “G. Martino” Hospital, University of Messina, 98124 Messina, Italy; silviabrunetto90@gmail.com (S.B.); francescadimasi5@gmail.com (F.D.); cristiano@imaiolo.it (C.M.); emanuela.zumbo.96@gmail.com (E.Z.); federicabuta@gmail.com (F.B.); gangemis@unime.it (S.G.)

**Keywords:** EGPA, ANCA, ANA, eosinophils, NLR, ESR, CRP, BVAS, early EGPA diagnosis

## Abstract

Eosinophilic granulomatosis with polyangiitis (EGPA) is a rare vasculitis. A key bio-logical hallmark is peripheral blood eosinophilia while antineutrophil cytoplasmic an-tibody (ANCA) positivity is not always present. Therefore, an early phase suspicion of EGPA leading to a correct diagnosis can be challenging. We performed an observa-tional cross-sectional study limited to baseline data on 38 patients between December 2019 and December 2025 at the Allergy and Clinical Immunology Unit, Messina, Italy. Data collection included eosinophil, neutrophil, and lymphocyte counts; ANCAs and antinuclear antibodies (ANAs); erythrocyte sedimentation rate (ESR) and C-reactive protein (CRP) determination; and the Birmingham vasculitis activity score (BVAS) questionnaire: Median eosinophil counts were 685 cells/µL, the neutro-phil-to-lymphocyte ratio (NLR) was 2.29, ESR median values were 24 mm/h, and CRPs were 1.99 mg/L. ANCAs were positive in five patients, and ANAs in 20. The trend in ANA+ patients was of lower median eosinophil counts and higher ESR and CRP me-dian values and median BVASs. General, nervous system, and ENT BVAS domains had higher scores.: Disease activity in EGPA at diagnosis can be difficult to recognize. We defined it as chameleon-like and could not be captured by specific immunological sig-natures. Therefore, an early diagnosis needs multidimensional assessment, integrating laboratory parameters with clinical scores. In our EGPA cohort, ANAs were detected in just over half the patients, suggesting that rather than acting as bystanders, these could help clinicians, together with eosinophil counts, NLRs, and BVASs, to suspect EGPA at an early stage for referral to specialized medical centers.

## 1. Introduction

Eosinophilic granulomatosis with polyangiitis (EGPA), formerly known as Churg–Strauss syndrome, is a rare systemic vasculitis [1]. It belongs to the group of antineutrophil cytoplasmic antibody (ANCA)-associated vasculitis (AAV) and is characterized by immune-mediated inflammation and the structural injury of vessels’ walls [2]. EGPA exhibits marked clinical heterogeneity, encompassing both eosinophil-mediated manifestations, such as asthma, recurrent pulmonary infiltrates, eosinophilic cardiomyopathy, and vasculitis features, including glomerulonephritis, mononeuritis multiplex, and purpura [3,4].

A key biological hallmark of EGPA is peripheral blood eosinophilia, even exceeding 1500 cells/µL (>10%), reflecting type 2 immune activation and eosinophil-driven tissue injury [5]. Elevated eosinophil counts have been consistently associated with asthma severity and cardiac involvement, underscoring their roles as both diagnostic and prognostic indicators [6]. A significant drawback is that eosinophilia can be significantly reduced or undetectable if the patient has been taking steroids and/or anti-inflammatory drugs [7].

Other laboratory findings should also be investigated when suspecting EGPA; the presence of ANCAs (ANCA+) delineates distinct phenotypic subsets of EGPA, but only approximately one third of patients are positive for perinuclear ANCAs (p-ANCAs), usually directed against myeloperoxidase (MPO) [8]. It is reported that ANCA+ patients generally exhibit a vasculitis phenotype, with higher frequencies of renal involvement, peripheral neuropathy, and increased disease activity scores [9]. In contrast, ANCA-negative (ANCA−) patients present more frequently with eosinophil-predominant disease [10]. Antinuclear antibodies (ANAs) can be present in EGPA patients [11] as in other autoimmune diseases [12], and it has been suggested that, instead of acting as bystanders, they may help clinicians to suspect EGPA [13].

Laboratory tests are obviously not enough on their own to diagnose EGPA, as clinical signs of systemic involvement must be investigated. The Birmingham vasculitis activity score (BVAS) is the most widely employed tool for quantifying disease activity in systemic forms of vasculitis, including EGPA, and considers nine domains that are differently represented in patients [14]. The BVAS domains are the general domain, which includes signs of autoimmune inflammation [15], as well as the nervous system [16]; ear, nose, and throat (ENT) [17]; cutaneous [13]; mucosal [18]; chest [19]; renal [20]; cardiovascular [21]; and abdominal [22] domains, which indicate the corresponding involvement of different organs and systems.

BVAS was initially validated for two other AAVs, granulomatosis with polyangiitis (GPA) and microscopic polyangiitis (MPA), and may underestimate the contribution of eosinophilic-driven organ involvement, which is central to EGPA pathophysiology and prognosis [23]. Nevertheless, it has been used for evaluating the response to biological treatments with anti-IL5 agents, Benralizumab and Mepolizumab [24,25].

Specific changes in complete blood count (CBC) components, such as eosinophil, neutrophil, and lymphocyte counts, are influenced by immune system responses to various factors, such as systemic inflammation; in EGPA, a complex interplay between eosinophilic inflammation and the clinical expression of the disease is described even if all the aspects of these interactions have not been explained [26].

The evaluation of both clinical manifestations and laboratory findings could provide critical insights into the spectrum of EGPA phenotyping to capture the full clinical burden of the disease [27].

The present study aims to analyze a single-center cohort of patients at EGPA diagnosis, who were followed at the Allergy and Clinical Immunology Unit of the G. Martino University Hospital of Messina, Italy.

The study searched for correlations between laboratory findings, such as peripheral blood eosinophil, neutrophil, and lymphocyte counts; ANCA and ANA serology; and inflammation markers, namely the erythrocyte sedimentation rate (ESR) and C-reactive protein (CRP), and clinical manifestations scored through the BVAS questionnaire. By correlating these laboratory and clinical parameters, we sought to find elements that might contribute to the early diagnosis of EGPA in order to distinguish and understand different disease activity patterns [28,29].

## 2. Results

ANCA positivity was present in 5/38 (13.2%) patients and ANA positivity in 20/38 (52.63%); the most common ANA patterns were fine speckled (AC-4) and homogeneous (AC-1). No significant association between ANCA and ANA positivity was observed (OR: 0.56, 95% CI: 0.07–4.45; *p* = 0.63, Fisher’s exact test).

Eosinophil, neutrophil, and lymphocyte counts, inflammatory markers (ESR and CRP), and BVAS results showed non-normal distributions in the overall cohort, appearing skewed with extreme values. Therefore, continuous data were summarized using median and IQR values (Table 1).

The neutrophil–lymphocyte ratio was also calculated (2.29 median, 1.65–2.81 IQR) and correlated with BVASs, but no significant correlation was observed (Spearman’s rho = 0.131, *p* = 0.432).

### 2.1. ANCA Status

Eosinophil counts, ESRs, CRPs, and BVASs were generally higher in ANCA+ patients compared to ANCA− patients. However, none of these differences reached statistical significance (eosinophils *p* = 0.172; ESR *p* = 0.604; CRP *p* = 0.167; BVAS *p* = 0.099) (Table 2).

### 2.2. ANA Status

Median eosinophil counts were slightly lower in ANA+ patients, but this difference did not reach statistical significance (*p*-value 0.099 > 0.05); ESR and CRP median values and median BVASs were higher in ANA+ patients but with no statistically significant differences (eosinophils *p* = 0.099; ESR *p* = 0.404; CRP = 0.464; BVAS = 0.848) (Table 3).

### 2.3. Results of Correlation Analysis

Considering data obtained from all the cohort of patients at EGPA diagnosis, correlation analysis did not reveal any significant association between BVAS results and eosinophil counts (Spearman’s ρ = 0.088, *p* = 0.599), nor between BVAS results and inflammatory markers (ESR ρ = 0.002, *p* = 0.991; CRP ρ = 0.051, *p* = 0.762). Similarly, eosinophil counts did not correlate with ESR (ρ = 0.02, *p* = 0.903) or CRP levels (ρ = 0.213, *p* = 0.200). A moderate and statistically significant positive correlation was observed between ESR and CRP (ρ = 0.506, *p* = 0.001), indicating internal consistency of systemic inflammatory markers (Table 4).

Analysis of BVAS domains at EGPA diagnosis showed that general (94.7%), nervous system (94.7%), and ENT (92.1%) were the most frequently involved, together with the chest (81.6%), mucosal (81.6%), and cutaneous (84.2%) domains. In contrast, renal (44.7%), cardiovascular (28.9%), and abdominal (21.1%) domains were less frequently involved (Table 5).

Analysis of the scores corresponding to each BVAS domain indicated the pattern and severity of organ involvement in our cohort of patients at EGPA diagnosis; nervous system (36/38) and ENT (35/38) manifestations were frequently present with median domain scores of five (IQR 4–8 and 4–7, respectively) indicating high symptoms. Chest (31/38) and mucous membranes/eyes (31/38) involvement had median domain scores of three (IQR 1–4 and 2.5–4, respectively), reflecting moderate symptom burden. A previous diagnosis of severe eosinophilic asthma was present in 24/38 patients at EGPA diagnosis, while 7/38 had been diagnosed with moderate eosinophilic asthma; patients with asthma were under treatment with either an association of inhaled corticosteroid + beta 2 long-acting agonist or, in addition to this, with a long-acting muscarinic antagonist.

General immune-rheumatologic manifestations (36/38) and abdominal (8/38) involvement both had median domain scores of two (IQR 2–4 and 2–2, respectively) with mild symptom burden. Cutaneous (32/38), renal (17/38), and cardiovascular (11/38) involvement were associated with low median scores of one (IQR 1–2, 1–2, and 1–2.5, respectively) indicating low symptom burden (Table 6).

The involvement of the different BVAS domains was comparable between ANCA+ and ANCA− patients except for the cardiovascular domain, which was observed only in the latter. As far as median and IQR BVAS values were concerned, no statistical analyses could be performed, as only five patients were ANCA+ and 33 patients were ANCA− overall (Table 7).

## 3. Discussion

EGPA is a rare vasculitis with a dual eosinophilic and vasculitis nature [30].

Recognition and diagnosis of EGPA is crucial and challenging [31], as nowadays, treatment initiation in the early phases of the disease with targeted precision medicines is a determinant for successful symptom control [32,33].

EGPA at diagnosis can be difficult to recognize, for which we defined it as chameleon-like, as it is not captured by specific immunological signatures; a marked heterogeneity in clinical presentation and laboratory findings can be present, underlying a complex and multifaceted immunopathogenesis which involves molecular and cellular pathways [34].

Eosinophilia, a hallmark of EGPA, is driven by several growth factors, including IL-3, IL-5, and granulocyte–macrophage colony-stimulating factor (GM-CSF), with IL-5 playing a central role in eosinophil proliferation, activation, and survival [35]. Elevated serum IL-5 levels have been consistently demonstrated in EGPA patients, and therapeutic targeting of this pathway has proven effective, underscoring its pathogenic relevance [36,37]. In addition, increased levels of soluble CD95 may further contribute to eosinophil accumulation by reducing eosinophil apoptosis [38]. While disease activity is amplified by eosinophils, immune-dysregulation is also enhanced by T-cell activation through IL-25 production, promoting both Th1/Th2 cytokine responses [39,40,41]. These alterations in regulatory and responder T-cell populations, particularly in reduced IL-10- and IL-2-producing T-cells, contribute to immune imbalance [42].

In our cohort of EGPA patients at diagnosis from a real-world setting, we explored the correlation between eosinophil, neutrophil, and lymphocyte counts, immunological features, inflammatory burden, and clinical manifestations to give a snapshot of patients’ characteristics at an early stage of diagnosis.

It has been reported that specific changes in complete blood count components, not just eosinophils but neutrophil and lymphocyte counts as well, are influenced by immune system responses causing systemic inflammation; specifically, the neutrophil-to-lymphocyte ratio (NLR) has shown a promising prediction in health conditions related to inflammation [43].

In AAV patients, an NLR cut-off value of 3.22 has been associated with reduced survival [44]. The median NLR in our EGPA cohort was 2.29, remaining below this threshold; only seven out of 38 patients (18.4%) had NLR values above three.

In our cohort at EGPA diagnosis, eosinophil counts did not correlate with inflammatory markers, such as ESR and CRP, nor with BVAS values; EGPA diagnosis therefore can be difficult when clinical disease activity is not directly reflected by peripheral eosinophilia or conventional inflammatory indices.

Stratification of our EGPA patients at diagnosis according to ANCA status revealed a trend toward higher eosinophil counts and increased CRP levels in ANCA+ patients; however, none of these differences reached statistical significance. Importantly, ANCA positivity was observed in only a very small number of patients (5/38), which substantially limited the statistical power of subgroup analyses and precluded definitive conclusions.

ANA positivity at EGPA diagnosis in our population did not identify a distinct inflammatory, eosinophilic, or clinical phenotype, even though this was frequently present; between ANA+ and ANA− patients, no relevant differences in eosinophil counts, inflammatory markers, or BVAS values were observed.

ANA positivity was frequent in this cohort, but its clinical significance requires further evaluation; in our opinion, ANA detection in the presence of increased eosinophil counts, excluding other causes of eosinophilia, such as parasitic infestation [41], could help clinicians to suspect EGPA.

BVAS domain analysis of our EGPA patients at diagnosis detailed organ involvement at disease onset, confirming multisystemic involvement [44]. The most frequently involved BVAS domains were the neurological, ENT, chest, mucocutaneous, and general domains, whereas renal, cardiovascular, and abdominal manifestations were less common. Stratification by ANCA status did not reveal clear differences in BVAS domain distribution; however, the marked imbalance between ANCA− and ANCA+ patients substantially limited interpretability. Notably, cardiovascular involvement was observed exclusively among ANCA− patients, a finding consistent with previous reports linking cardiac manifestations to ANCA-negative, eosinophil-driven disease [45]. BVAS domains with higher scores and therefore higher symptom burdens were the general, nervous system, and ENT domains, as reported in the literature [16,46].

BVAS is a reliable tool to investigate clinical manifestations of AAV, but the exclusive reliance on BVAS for EGPA diagnosis may underestimate crucial aspects of the disease: eosinophilia [44], and patients’ perceived physical health [47]. A weighted model, such as a BVAS-EGPA questionnaire, could help bridge this gap by providing a more accurate reflection of real-world disease activity and improving patient stratification. Future multi-center and retrospective studies will be essential to validate an adapted BVAS-EGPA diagnostic tool and to test its predictive performance regarding clinical outcomes and treatment response.

The AAV patient-related outcomes (AAV-PRO) questionnaire, as it evaluates not just organ-specific symptoms but physical function also, has been proven useful in evaluating the quality of life of EGPA patients [32].

### Strengths and Limitations

This study has several limitations. One is the possible residual steroid effect on eosinophil counts, as all patients started OCS weaning, from a mean dose of 25.66 mg/day, only one week before blood tests.

Other limitations were the relatively small sample size and the very limited number of ANCA-positive patients, which restrict the ability to draw firm conclusions from subgroup analyses. In addition, due to the sample size and the number of comparisons performed, the results should be cautiously interpreted as exploratory.

Nevertheless, the comprehensive evaluation of clinical, laboratory, and immunological features at diagnosis provides a meaningful descriptive snapshot of EGPA heterogeneity and supports the hypothesis of distinct disease phenotypes that cannot be fully defined by autoantibody status alone. Larger, prospective studies are warranted to identify reliable biomarkers for disease stratification, eventually even for identifying different clusters of disease, and different treatment approaches [48].

## 4. Materials and Methods

An observational study was performed on 38 patients, comprising 28 females (73.68%) and 10 males (26.32%), aged 56 ± 15 years, at EGPA diagnosis according to ACR/EULAR criteria [49]. Although the study was designed with prospective data collection, analysis was conducted on baseline data and is therefore cross-sectional. Participants were recruited between December 2019 and December 2025, consecutively, at the Allergy and Clinical Immunology Unit of the G. Martino University Hospital of Messina, Italy. All participants provided written informed consent before inclusion in the study after being informed about the research objectives, privacy protection, and the use of anonymous data, in compliance with the European General Data Protection Regulation (GDPR) 2016/679. The study was approved by the Ethics Committee of the G. Martino University Hospital of Messina, Italy (protocol number 16/19) on 18 March 2019.

Data collection included a complete blood count to evaluate eosinophil, neutrophil and lymphocyte values, ANCA and ANA determination, and the dosage of inflammatory markers, such as ESR and CRP; the BVAS questionnaire was also submitted to all patients.

All patients underwent oral corticosteroid weaning to stop steroid treatment one week before blood tests.

To exclude other causes of eosinophilia other than EGPA, all patients underwent a parasitic stool examination and a peripheral blood smear, and were only included in the study if both tests were negative.

CRP alterations were further investigated by measuring procalcitonin blood levels, which were negative in all patients.

### Statistical Analysis

Continuous variables, such as blood eosinophil, neutrophil, and lymphocyte counts; ESRs; CRPs; and BVAS values, were examined for their distributional characteristics using the Shapiro–Wilk test. Data, as not normally distributed, were summarized using median and interquartile ranges (IQRs). Categorical variables were reported as absolute numbers and percentages.

Comparisons between groups were performed using non-parametric tests; differences in continuous variables between ANCA+ and ANCA− patients, as well as ANA+ and ANA− patients, were assessed using the Mann–Whitney U test.

Associations between categorical variables, such as ANCA and ANA positivity, were evaluated using Fisher’s exact test.

Correlations between eosinophil counts, inflammatory markers (ESR and CRP), and BVAS values were calculated using Spearman’s rank correlation coefficient (ρ). The same correlation was applied for evaluating the neutrophil-to-lymphocyte ratio (NLR).

All statistical analyses were performed using the Jamovi statistical software (version 2.7.15). Given the relatively small sample size and multiple comparisons, the analyses are to be considered exploratory. A *p*-value < 0.05 was considered statistically significant.

## 5. Conclusions

Our findings provide a comprehensive description of immunological and inflammatory features of EGPA at diagnosis and emphasize that disease activity cannot be adequately captured by single laboratory parameters. Rather, EGPA encompasses heterogeneous and partially overlapping pathogenic pathways, with a “chameleon-like” subdual appearance due to the diverse clinical phenotypes at presentation. A multidimensional assessment of EGPA patients, integrating clinical scores collected through the BVAS questionnaire, general laboratory findings, and immunological features, remains essential and can facilitate an early diagnostic suspicion, helping with referrals to specialized medical centers.

## Figures and Tables

**Table 1:** Continuous variables present in EGPA patients at diagnosis, such as eosinophils, neutrophils, lymphocytes, ESRs, CRPs, and BVASs, are described and summarized using median and IQR values.

Variable	Value
Number of patients	38
ANCA positivity, n (%)	5 (13.2%)
ANA positivity, n (%)	20 (52.63%)
Eosinophils (cells/µL), median (IQR)	685 (504–1296)
Neutrophils (cells/µL), median (IQR)	4470 (3482.75–5796.5)
Lymphocytes (cells/µL), median (IQR)	2067 (1801–2382)
ESR (mm/h), median (IQR)	24 (4.25–44.5)
CRP (mg/L), median (IQR)	1.99 (0.17–3.8)
BVAS, median (IQR)	18.5 (15–21.75)

**Table 2:** Median values of eosinophil count, ESR, CRP, and BVAS results in ANCA+ and ANCA− patients.

Variable	ANCA+	ANCA–	*p*-Value
Eosinophils (cells/µL), median (IQR)	1470 (960–6048)	630 (504–1092)	0.172
ESR (mm/h), median (IQR)	28 (27–35)	15 (4–45)	0.604
CRP (mg/L), median (IQR)	2.87 (1.9–9.85)	1.6 (0.16–3.8)	0.167
BVAS, median (IQR)	26 (18–26)	18 (15–20)	0.099

**Table 3:** Median values of eosinophil counts, ESRs, CRPs, and BVASs in ANA+ and ANA− patients at EGPA diagnosis.

Variable	ANA+ (n = 20)	ANA– (n = 18)	*p*-Value
Eosinophils (cells/µL), median (IQR)	565 (470–993)	877.5 (612.75–1446.5)	0.099
ESR (mm/h), median (IQR)	27 (4.75–45.75)	18.5 (3.5–28)	0.404
CRP (mg/L), median (IQR)	1.11 (0.17–2.84)	0.46 (0.23–3.71)	0.464
BVAS, median (IQR)	19 (15–21.25)	17 (15–21.5)	0.848

**Table 4:** Results of correlation analyses between different parameters evaluated in EGPA patients at diagnosis: BVAS, ESR, and CRP vs. eosinophils; BVAS vs. ESR and CRP; ESR vs. CRP.

Variable	*ρ* (Spearman)	*p*-Value	
BVAS vs. eosinophils	0.088	0.599	No significant correlation
ESR vs. eosinophils	0.02	0.903	
CRP vs. eosinophils	0.213	0.200	
BVAS vs. ESR	0.002	0.991	
BVAS vs. CRP	0.051	0.762	
ESR vs. CRP	0.506	0.001	Positive and statistically significant correlation

**Table 5:** Description of BVAS domains in our cohort of EGPA patients at diagnosis.

BVAS Domains	N (%) of EGPA Patients at Diagnosis
General (arthritis, muscle aches, fever)	36 (94.7%)
Nervous system (sensory peripheral neuropathy)	36 (94.7%)
ENT (paranasal sinus involvement, chronic rhinosinusitis with nasal polyps)	35 (92.1%) 20 (52.6%)
Cutaneous (skin vasculitis/purpura)	32 (84.2%)
Mucous membranes/eyes (mouth ulcers, blurred vision)	31 (81.6%)
Chest (wheeze, infiltrates)	31 (81.6%)
Renal (hypertension, proteinuria, ematuria)	17 (44.7%)
Cardiovascular (valvular heart diseases, pericarditis, cardiomyopathy)	11 (28.9%)
Abdominal (ischemic abdominal pain, bloody diarrhea)	8 (21.1%)

**Table 6:** Median and IQR scores of BVAS domains at EGPA diagnosis; higher median scores indicate a higher symptom burden.

BVAS Domain	N (>0)	Median Score	IQR
General	36	2	2–4
Nervous system	36	5	4–8
ENT	35	5	4–7
Cutaneous	32	1	1–2
Mucous membranes/eyes	31	3	2.5–4
Chest	31	3	1–4
Renal	17	1	1–2
Cardiovascular	11	1	1–2.5
Abdominal	8	2	2–2

**Table 7:** Median and IQR scores of BVAS domains, stratified according to ANCA status in EGPA patients at diagnosis.

BVAS Domain	ANCA− Median (IQR)	ANCA+ Median (IQR)
General	2 (IQR 2–3)	2 (IQR 2–4)
Nervous system	5 (IQR 3–7)	7 (IQR 5–8)
ENT	5 (IQR 3–7)	4 (IQR 4–4)
Cutaneous	1 (IQR 1–2)	2 (IQR 1–2)
Mucous membranes/eyes	3 (IQR 1–4)	3 (IQR 2–4)
Chest	2 (IQR 1–3)	3 (IQR 2–5)
Renal	0 (IQR 0–1)	1 (IQR 1–1)
Cardiovascular	0 (IQR 0–1)	0 (IQR 0–0)
Abdominal	0 (IQR 0–0)	0 (IQR 0–0)

## Data Availability

Data are available upon reasonable request.

## Abbreviations

The following abbreviations are used in this manuscript:

EGPA	Eosinophilic granulomatosis with polyangiitis
ANCA	Antineutrophil cytoplasmic antibody
ANA	Antinuclear antibody
ESR	Erythrocyte sedimentation rate
CRP	C-reactive protein
BVAS	Birmingham vasculitis activity score
ENT	Ear, nose, and throat
AAV	ANCA-associated vasculitis
MPO	Myeloperoxidase
MPA	Microscopic polyangiitis
GPA	Granulomatosis with polyangiitis
OR	Odds ratio
IQR	Interquartile range
GM-CSF	Granulocyte-macrophage colony-stimulating
AAV-PRO	ANCA-associated vasculitis patient-related outcome
